# Association between HLA-DRB1*04:05 and the efficacy of immune checkpoint inhibitors for patients with advanced cancer

**DOI:** 10.3389/fendo.2026.1789039

**Published:** 2026-06-10

**Authors:** Mayu Watanabe, Jun Eguchi, Atsushi Takamoto, Jun Wada

**Affiliations:** 1 Department of Diabetology and Endocrinology, NHO Okayama Medical Center, Okayama, Japan; 2 Department of Nephrology, Rheumatology, Endocrinology and Metabolism, Okayama University Faculty of Medicine, Dentistry and Pharmaceutical Sciences, Okayama, Japan; 3 Department of Urology, Fukuyama City Hospital, Hiroshima, Japan

**Keywords:** anti-PD1 immune checkpoint inhibitors, human leukocyte antigen class II alleles, progression-free survival, treatment response, type 1 diabetes

## Abstract

**Introduction:**

Tumor cells use immune checkpoint proteins such as programmed cell death 1 to escape immunological defenses. Immune checkpoint inhibitors (ICIs) that target these proteins have been used to treat several malignancies. ICI-induced diabetes is a rare but life-threatening adverse event. Our previous study evaluated pancreatic β-cell activity using the homeostasis model assessment of β-cell (HOMA-β) function before ICI treatment and demonstrated its association with treatment outcomes. In addition, DRB1*04:05 and DRB1*09:01 reportedly increase the risk of type 1 diabetes in Japanese patients, whereas DRB1*15:01 protected against type 1 diabetes. However, the association between human leukocyte antigen (HLA) class II alleles and treatment outcomes of ICI therapy remains unclear.

**Methods:**

We included 96 patients who were diagnosed with advanced cancer. The HLA genotypes that cause type 1 diabetes in patients with ICI-treated cancers were evaluated to determine their association with the cancer prognosis.

**Results:**

The median progression-free survival (PFS) in the DRB1*04:05-positive group (2 months, 95% confidence interval [CI]: 1.214–2.786; 31 events; 1 censored event) was significantly shorter than that in the DRB1*04:05-negative group (3 months; 95% CI: 1.933–4.067; 56 events; 8 censored events) (log rank p=0.045). Additionally, a multivariable Cox proportional hazards regression revealed that DRB1*04:05 was independently associated with shorter PFS for patients treated with ICIs.

**Conclusions:**

HLA class II alleles were associated with shorter PFS in patients treated with ICIs. In particular, DRB1*04:05 positivity was associated with worse survival outcomes, suggesting a potential immunogenetic contribution to treatment outcomes.

## 1 Introduction

Tumor cells inhibit immune responses through immune checkpoint proteins such as programmed cell death 1 (PD-1) to escape immunological defenses. Immune checkpoint inhibitors (ICIs) that target these proteins are effective against numerous malignancies (1). However, ICI administration is known to induce various immune-related adverse events, including autoimmune diabetes (2). Moreover, because ICIs may not guarantee efficacy or survival extension in all patients, the identification of biomarkers associated with treatment outcomes has emerged as a significant issue (3).

We previously conducted a homeostasis model assessment of β-cell (HOMA-β) function and reported that the pancreatic β-cell function before ICI treatment is associated with overall survival (OS) (4). These findings indicate that the capacity to tolerate immune-mediated stress, reflected in β-cell function, may influence treatment outcomes. Furthermore, genetic background and especially the human leukocyte antigen (HLA) genotype have been associated with the risk of ICI-induced diabetes and the response to ICI therapy (5). HLA class II molecules play a central role in antigen presentation and activation of CD4+ T cells, which are essential for orchestrating anti-tumor immune responses. In addition, HLA polymorphisms influence susceptibility to autoimmunity, which may further modulate immune-related responses during ICI therapy. Although numerous genes influence susceptibility to type 1 diabetes (T1D), HLA-DR and HLA-DQ genes are especially significant (6, 7). In Japanese individuals, the haplotypes DRB1*04:05-DQB1*0401 and DRB1*09:01-DQB1*0303 are associated with an increased risk of T1D; however, DRB1*15:01-DQB1*06:02 has a protective effect against T1D (8–10). The HLA genotype is involved in the onset of T1D, which is characterized by depleted insulin secretion. Because HLA class II alleles regulate autoimmune responses against pancreatic β cells, HLA genotype may indirectly influence ICI efficacy through its effects on β-cell vulnerability and immune regulation. However, few studies have specifically examined the association between HLA class II alleles and treatment outcomes of ICI therapy, and their role in treatment outcomes remains unclear. Therefore, we sought to investigate the association between HLA genotype and the efficacy of ICIs.

The principal aim of this study was to assess the association between HLA class II alleles and treatment outcomes in patients receiving ICI therapy. Furthermore, we evaluated the association between HLA polymorphisms and changes in glucose tolerance before and after ICI administration.

## 2 Materials and methods

### 2.1 Study participants

The present study included a cohort of 96 individuals diagnosed with advanced cancer who were recruited between June 2017 to August 2019. The recruitment timeframe, inclusion criteria for patients with advanced cancer, study design, and clinical setting were consistent with those described in our previous study (4). In this prospective cohort, 104 patients were initially enrolled; however, eight patients were excluded for the following reasons: unknown HLA-DRB1 status (n=5), withdrawal of consent (n=1), transfer to another hospital within 1 month after the initial ICI administration (n=1), and receipt of an anti-PD-L1 antibody (n=1). Finally, 96 patients were included in this study. Among the included individuals, 83 overlapped with those in our previous study that investigated the association between β-cell function and OS of patients receiving ICIs (4). This study was an independent investigation that focused on analyzing the association between HLA class II alleles and treatment outcomes following ICI treatment, and it included additional analyses of changes in glucose metabolism before and after treatment. All participants in this study were of Japanese ethnicity and received at least one dose of an intravenous anti-PD1 drug (nivolumab or pembrolizumab) as initial immunotherapy. Participants in this study did not receive concomitant steroid therapy or chemotherapy during the observation period. The prognosis was monitored until September 2022. Written informed consent was obtained from all patients. The study protocol was approved by the Ethics Committee of Okayama University (1704-009) and conducted in accordance with the Declaration of Helsinki.

### 2.2 Data collection

Data were prospectively collected in accordance with the protocol of our previously reported cohort study (4). Data collection and management methods were consistent with those described in that study and are briefly summarized in this report. HLA-DRB1 and HLA-DQB1 alleles were determined using next-generation sequencing sequence-based typing performed by a certified external laboratory (SRL, Inc., Tokyo, Japan), with a typing resolution of six digits.

### 2.3 Definitions

Clinical variables and outcomes were defined as previously reported (4). Tumor response was assessed using the Response Evaluation Criteria in Solid Tumors (RECIST version 1.1) (11). Progression-free survival (PFS) and OS were used as outcome measures. OS was defined as the time from initiation of the first ICI administration to death from any cause. PFS was defined as the time from initiation of the first ICI administration to disease progression or death from any cause. Prognosis was monitored until September 2022. Tumor assessment schedules were not standardized because of differences in cancer types and clinical practice; however, all available imaging data were collected and used for analysis. For OS, patients who were alive at the end of follow-up were censored at the date of their last clinical visit. For PFS, patients alive without disease progression were censored at the date of their most recent imaging assessment. Central review of imaging was not performed.

### 2.4 Statistical analysis

Based on prior evidence regarding type 1 diabetes susceptibility and resistance in the Japanese population, we pre-specified three candidate HLA-DRB1 alleles (DRB1*04:05, DRB1*09:01, and DRB1*15:01) for analysis (8–10). No additional HLA-DRB1 alleles were tested beyond these pre-specified candidates.

The Mann–Whitney U test was used to evaluate differences in HbA1c, C-peptide index, β-cell function (HOMA-β), and insulin resistance (homeostatic model assessment of insulin resistance [HOMA-IR]) before and after ICI administration. Differences in C-reactive protein (CRP), and creatinine according to HLA-DRB1 alleles were also evaluated. The association between HLA-DRB1 alleles and cancer types was analyzed using the chi-square test. Cancer type was categorized into three groups: head and neck cancer, gastric cancer, and other malignancies, and included as a categorical variable in the Cox proportional hazards models. PFS and OS since the date of the first treatment and survival curves were generated using the Kaplan–Meier method and compared using the log-rank test. Univariate and multivariate Cox proportional hazards models were used to estimate hazard ratios for the covariates. The proportional hazards assumption was assessed by including time-dependent covariates in the Cox models. Cases with missing covariate data were excluded from the Cox regression analyses. Missing data were observed for Eastern Cooperative Oncology Group performance status (ECOG PS) (n=4) and CRP (n=1); therefore, both univariable and multivariable Cox regression analyses were performed using complete-case data (n=91). No missing data were observed for the other covariates included in the analysis. All statistical analyses were performed using SPSS version 26 (IBM Corp., Armonk, NY, USA). Statistical significance was set at p < 0.05.

## 3 Results

### 3.1 Baseline characteristics of the study participants

This study included 96 participants (64 male and 32 female patients). The baseline characteristics of the study participants are shown in **Table 1**. The participants had a median age of 65 years and median body mass index (BMI) of 19.4 kg/m^2^. Among the participants, 32 tested positive for DRB1*04:05, and all of these individuals also tested positive for DQB1*04:01. Similarly, 16 individuals had DRB1*09:01, and all of these individuals also exhibited DQB1*03:03; however, eight individuals had DRB1*15:01, and all of them also had DQB1*06:02.

**Table 1:** Baseline characteristics of the study participants.

	All patients (n=96)
Age (years)	65 (57–73)
Sex (Male)	64
BMI (kg/m^2^)	19.4 (17.8–21.6)
PFS (m)	2.5 (1.0–5.0)
OS (m)	8.0 (3.0–20.3)
HLA-DRB1 (n)	
DRB1^*^04:05	32
DRB1^*^09:01	16
DRB1^*^15:01	8
ICI (n)	
Nivolumab	84
Pembrolizumab	13
Ipilimumab	2
Primary tumor (n)	
Head and neck cancer	56
Gastric cancer	22
Genitourinary cancer	15
Other	3
ECOG-PS	
0–1	78
≥2	14
History of diabetes, n	12
Laboratory data	
CRP (mg/dL)	0.39 (0.18–2.25)
Cr (mg/dL)	0.76 (0.64–0.91)
Glucose metabolism markers	
HbA1c (%)	5.6 (5.3–5.9)
Fasting plasma glucose (mg/dL)	98 (88–111)
C-peptide index	1.57 (1.10–2.15)
HOMA-β	51.2 (36.5–83.8)
HOMA-IR	1.14 (0.73–2.29)

Data are shown as median (25–75th percentile) for continuous variables and as the number of cases (n) for categorical variables.

BMI, body mass index; OS, overall survival; PFS, progression–free survival; ICI, immune checkpoint inhibitor; ECOG PS, Eastern Cooperative Oncology Group performance status; CRP, C–reactive protein; HbA1c, glycated hemoglobin; FPG, fasting plasma glucose; CPI, C–peptide index; HOMA–β, homeostasis model assessment of beta cells; HOMA–IR, homeostasis model assessment of insulin resistance.

Among the participants, 84 received nivolumab, 13 received pembrolizumab, and 2 received ipilimumab. One patient received nivolumab and ipilimumab after pembrolizumab therapy, and another patient received nivolumab in combination with ipilimumab. We observed a total of 56 cases of head and neck cancer, 22 of gastric cancer, 15 of genitourinary cancer, and 3 of various other types of cancer (specifically, two cases of colon cancer and one case of malignant melanoma). No substantial correlation between the presence of HLA-DRB1*04:05, HLA-DRB1*09:01, or HLA-DRB1*15:01 and various cancer types, as determined using the Chi-square test (data not shown), was observed. Furthermore, 78 patients had an ECOG PS of 0 or 1. The laboratory results indicated no signs of renal impairment.

### 3.2 Association of HLA class II alleles with the cancer prognosis

**Figure 1:**
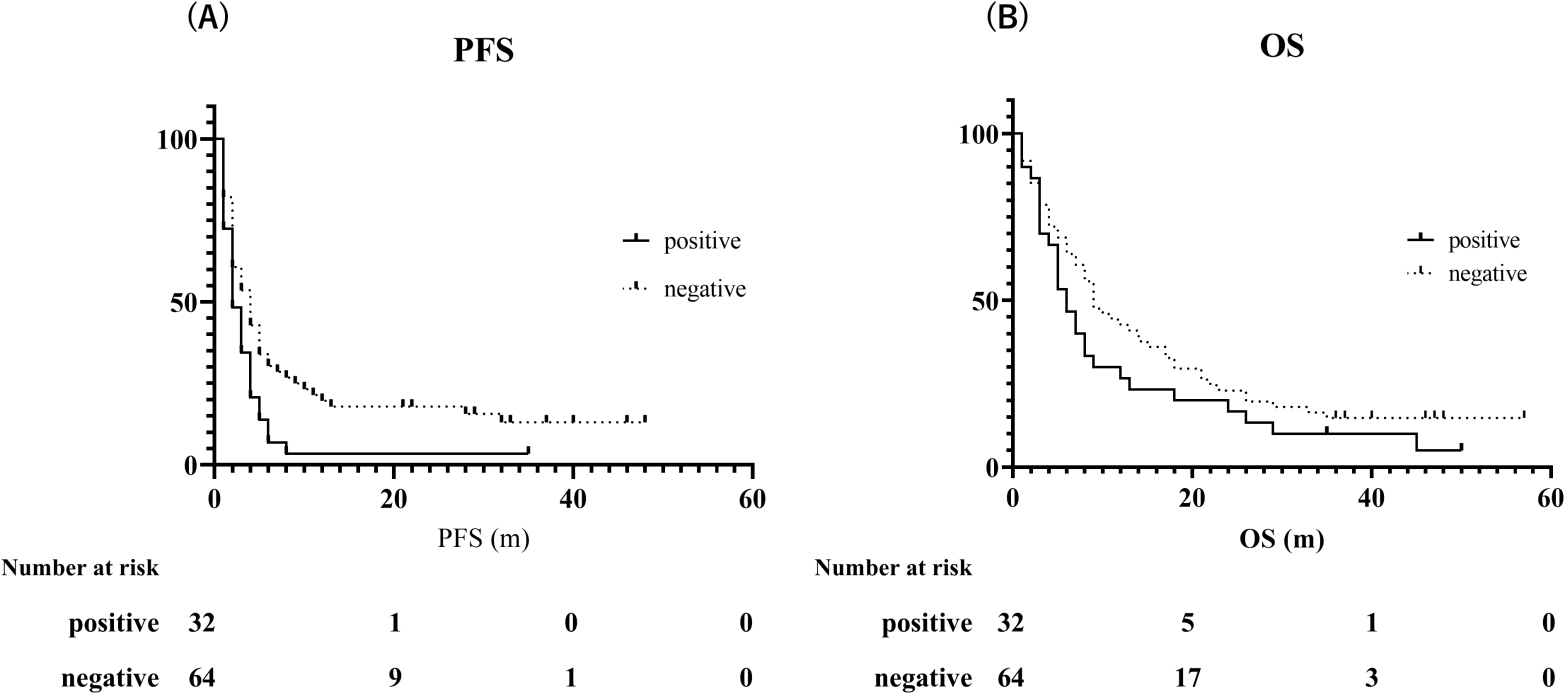
**(A)** Kaplan–Meier curves of progression-free survival (PFS) of patients with the presence or absence of DRB1*04:05. **(B)** Kaplan–Meier curves of overall survival (OS) of patients with the presence or absence of DRB1*04:05.

A total of 87 PFS events were observed in the study population. In Kaplan–Meier analysis, the median PFS in the DRB1*04:05-positive group (2 months; 95% confidence interval [CI]: 1.214–2.786; 31 events; 1 censored event) was significantly shorter than that in the DRB1*04:05-negative group (3 months; 95% CI: 1.933–4.067; 56 events; 8 censored events) (log-rank p=0.045) (**Figure 1A**). In the Kaplan–Meier analysis of the full cohort (n=96), the 3-month PFS rate was lower in the DRB1*04:05-positive group (31.3%) than in the negative group (46.9%).

A total of 85 OS events were observed. In Kaplan–Meier analysis, although not statistically significant, the median OS in the DRB1*04:05-positive group (5 months; 95% CI: 3.152–6.848; 30 events; 2 censored events) was shorter than that in the DRB1*04:05-negative group (9 months; 95% CI: 6.398–11.602; 55 events; 9 censored events) (log-rank p=0.145) (**Figure 1B**).

**Table 2** presents the baseline clinical characteristics according to DRB1*04:05 status. PFS and OS are presented as descriptive statistics only and were not used for statistical comparisons. All group comparisons for PFS and OS were performed using Kaplan–Meier analysis with the log-rank test and Cox proportional hazards models. Baseline characteristics were generally comparable between the groups, as indicated by the small standardized mean differences for most variables (**Table 2**). We further examined the association between DRB1*04:05 positivity and PFS or OS using multivariable Cox proportional hazards regression models to estimate hazard ratios, adjusting for age, sex, BMI, ECOG PS (0–1 vs ≥2), cancer type, and treatment regimen. DRB1*04:05 positivity was independently associated with shorter PFS (hazard ratio [HR], 1.640; 95% CI, 1.017–2.645; p = 0.043). Age, BMI, and CRP were also significantly associated with PFS in the multivariable analysis (**Table 3**).

**Table 2:** Baseline clinical characteristics according to DRB1*04:05 status.

	DRB1*04:05 negative (n=64)	DRB1*04:05 positive (n=32)	P–value	SMD
Age (years)	65 (57–72)	66 (56–75)	0.957	0.09
Sex (male, n, %)	43 (67.2%)	21 (65.6%)	0.878	0.27
BMI (kg/m^2^)	19.5 (17.5–21.5)	19.4 (17.9–22.2)	0.994	0.08
PFS (m)	3 (1.933–4.067)	2 (1.214–2.786)		
OS (m)	9 (6.398–11.602)	5 (3.152–6.848)		
Primary tumor (n, %)				
Head and neck cancer	34 (53.1%)	22 (68.8%)	0.143	0.12
Gastric cancer	16 (25.0%)	6 (18.8%)	0.492	0.03
Genitourinary cancer	11 (17.2%)	4 (12.5%)	0.767	0.10
ICI (n, %)				
Nivolumab	55 (85.9%)	29 (90.6%)	0.513	0.14
Pembrolizumab	10 (15.6%)	3 (9.4%)	0.534	0.20
Ipilimumab	2 (3.1%)	0 (0%)	0.551	0.18
ECOG PS ≥2 (n, %)	9 (14.1%)	5 (15.6%)	0.937	0.08
Laboratory data				
CRP (mg/dL)	0.53 (0.18–3.24)	0.34 (0.18–1.53)	0.429	0.05
Cr (mg/dL)	0.76 (0.64–0.90)	0.76 (0.66–0.94)	0.876	0.14
Glucose metabolism markers				
HbA1c (%)	5.6 (5.3–5.9)	5.7 (5.3–6.0)	0.564	0.22
Fasting plasma glucose (mg/dL)	98 (90–111)	96 (85–109)	0.311	0.27
C–peptide index	1.57 (1.05–2.03)	1.64 (1.18–2.43)	0.565	0.11
HOMA–β	45.6 (29.1–78.8)	64.0 (41.1–102.3)	0.057	0.39
HOMA–IR	1.07 (0.65–2.26)	1.39 (0.81–2.78)	0.304	0.12
ICI related diabetes mellitus	2	0	0.551	0.00

Data are shown as median (25–75th percentile) for continuous variables and as the number of cases (n) for categorical variables. Categorical variables are presented as number (percentage).

P-values were calculated using the Mann–Whitney U test for continuous variables and the χ² test or Fisher’s exact test for categorical variables.

PFS and OS are presented as median survival time (95% confidence interval) estimated using the Kaplan–Meier method and were not used for statistical comparisons.

SMD, standardized mean difference; values <0.1 indicate negligible differences. SMDs were calculated based on means and standard deviations.

BMI, body mass index; CRP, C-reactive protein; ECOG PS, Eastern Cooperative Oncology Group performance status; FPG, fasting plasma glucose; HbA1c, glycated hemoglobin; CPI, C-peptide index; HOMA-β, homeostasis model assessment of beta cells; HOMA-IR, homeostasis model assessment of insulin resistance; ICI, immune checkpoint inhibitor; OS, overall survival; PFS, progression-free survival.

**Table 3:** Cox proportional–hazards regression: univariate and multivariate analyses of progression–free survival (PFS).

	Univariate analysis			Multivariate analysis		
	HR	95% CI	P–value	HR	95% CI	P–value
DRB1^*^04:05 positive	1.389	0.885–2.178	0.153	1.640	1.017–2.645	0.043
Age (years)	0.993	0.974–1.012	0.456	0.981	0.962–1.000	0.049
Sex (male)	0.998	0.631–1.578	0.992	0.955	0.583–1.562	0.854
ECOG PS (≥2 vs 0–1)	1.640	0.914–2.943	0.097	1.312	0.677–2.543	0.422
BMI (kg/m²)	0.938	0.879–1.000	0.050	0.926	0.863–0.995	0.036
CRP (mg/dL)	1.125	1.059–1.195	<0.001	1.129	1.061–1.200	<0.001
Cancer type						
Gastric vs head and neck	1.176	0.700–1.975	0.541	1.448	0.797–2.631	0.224
Other vs head and neck	0.782	0.431–1.419	0.418	0.858	0.301–2.449	0.775
Treatment regimen						
Other vs nivolumab monotherapy	1.277	0.656–2.485	0.471	0.710	0.214–2.350	0.575

Multivariate analysis: independent variables were age, sex (male), BMI, ECOG PS, CRP, cancer type, treatment regimen and DRB1^*^04:05 positivity. BMI, body mass index; ECOG PS, Eastern Cooperative Oncology Group performance status; CRP, C–reactive protein; HR, hazard ratio; CI, confidence interval.

To further address clinical heterogeneity, we performed sensitivity analyses in more homogeneous subsets. In the sensitivity analysis restricted to patients with head and neck cancer, DRB1*04:05 positivity remained directionally consistent with the main analysis for PFS (HR 1.262, 95% CI 0.702–2.269, p=0.437). Similarly, in the analysis restricted to patients receiving nivolumab monotherapy, the direction of the association for PFS was unchanged (HR 1.472, 95% CI 0.899–2.409, p=0.124).

The proportional hazards (PH) assumption was assessed using time-dependent covariates. No significant violation of the PH assumption was observed for DRB1*04:05, sex, cancer type, treatment regimen, ECOG performance status, or C-reactive protein. However, significant time-dependent effects were observed for age (p < 0.001) and BMI (p = 0.031), suggesting potential departures from the PH assumption for these covariates.

In the adjusted multivariable Cox proportional hazards analysis, DRB1*04:05 positivity was independently associated with shorter OS (HR, 1.792; 95% CI, 1.088–2.953; p = 0.022). In addition, cancer type was significantly associated with OS (**Table 4**).

**Table 4:** Cox proportional–hazards regression: univariate and multivariate analyses of overall survival (OS).

	Univariate analysis			Multivariate analysis		
	HR	95% CI	P–value	HR	95% CI	P–value
DRB1^*^04:05 positive	1.357	0.861–2.137	0.188	1.792	1.088–2.953	0.022
Age (years)	1.016	0.995–1.037	0.135	1.009	0.989–1.029	0.392
Sex (male)	1.240	0.775–1.982	0.370	1.412	0.845–2.359	0.188
ECOG PS (≥2 vs 0–1)	2.796	1.524–5.131	0.001	1.559	0.790–3.074	0.200
BMI (kg/m^2^)	0.910	0.847–0.978	0.010	0.882	0.814–0.955	0.002
CRP (mg/dL)	1.123	1.057–1.193	<0.001	1.154	1.080–1.233	<0.001
Cancer type						
Gastric vs head and neck	1.874	1.110–3.165	0.019	2.789	1.506–5.165	0.001
Other vs head and neck	0.966	0.522–1.789	0.912	1.142	0.341–3.820	0.829
Treatment regimen						
Other vs nivolumab monotherapy	1.052	0.542–2.042	0.882	0.409	0.108–1.547	0.188

Multivariate analysis: independent variables were age, sex (male), BMI, ECOG PS, CRP, cancer type, treatment regimen and DRB1^*^04:05 positivity. BMI, body mass index; ECOG PS, Eastern Cooperative Oncology Group performance status; CRP, C–reactive protein; HR, hazard ratio; CI, confidence interval.

No significant associations with PFS or OS were observed for other HLA class II alleles, including DRB1*09:01 and DRB1*15:01.

### 3.3 Association of HLA class II alleles with diabetes onset

No association was noted between HLA class II alleles and diabetes onset. We analyzed alterations in glucose metabolism markers from before to 1 month after ICI administration based on the presence or absence of T1D-susceptible HLA class II alleles. After excluding individuals who received anti-diabetic medication and those with glycemic values in the diabetic range at baseline, HbA1c in 72 patients and fasting blood parameters in 52 patients were assessed. Only limited alterations in glucose metabolism were observed in relation to T1D-susceptible HLA class II alleles. Compared with DRB1*09:01-negative patients, DRB1*09:01-positive patients exhibited a modest yet statistically significant increase in HbA1c at 1 month after ICI administration. No significant differences in other metabolic parameters, including fasting plasma glucose, C-peptide index, β-cell function (HOMA-β), or insulin resistance (HOMA-IR) (**Supplementary Table 1**), were observed. Similarly, the DRB1*04:05 status and DRB1*15:01 status were not associated with any significant alterations in glycemic parameters (**Supplementary Table 2** and **3**).

## 4 Discussion

We investigated the effects of HLA class II alleles on treatment outcomes in patients receiving ICI therapy, with a primary focus on PFS. We found that DRB1*04:05 positivity was associated with shorter PFS. In addition, multivariable Cox analysis showed an association with shorter OS, although this finding should be interpreted with caution given tumor heterogeneity.

HLA class II molecules play a central role in antigen presentation and activation of CD4+ T cells (12). Each protein antigen comprises several epitopes that can be identified by different HLA class II molecules. The number of epitopes and their corresponding binding affinities for each HLA allele vary. Thus, the immune response elicited against the identical antigen differs based on HLA allele polymorphism (13). Systemic immune activation is critical for tumor elimination in cancer immunotherapy. While several therapies enhance the cytotoxic activity of CD8+ T cells, CD4+ T cells also play a crucial role in orchestrating anti-tumor immune responses. HLA class II–restricted CD4+ T cells recognize tumor antigens presented by antigen-presenting cells and contribute to immune activation through cytokine production and interaction with effector cells (14–16). These findings are consistent with a potential role of HLA class II–restricted immune responses in shaping ICI treatment outcomes and may provide a possible explanation for the observed association with PFS.

Previous studies have reported associations between particular HLA alleles and clinical outcomes of ICI therapy (17). Improved survival has been reported in carriers of HLA-DRB4 and HLA-A*01 in predominantly White populations (18, 19). HLA polymorphisms vary across populations (20), and associations observed in one population may not apply to others (21). In Japanese individuals, the DRB1*04:05-DQB1*0401 haplotype is relatively prevalent and is associated with susceptibility to type 1 diabetes (22). Therefore, the association between DRB1*04:05 and shorter PFS observed herein may reflect population-specific HLA distributions and partly explain the differences from previous reports.

The potential influence of tumor type on survival cannot be excluded. Previous studies examining HLA and prognosis within specific cancers are limited. For example an increased prevalence of the HLA-DR4 antigen has been reported in long-term survivors of gastric cancer (23), and an association between nasopharyngeal carcinoma and HLA-A*0207 has been observed in the Chinese population (24). In our study, sensitivity analyses restricted to more homogeneous subsets, including patients with head and neck cancer, showed that the direction of the association between DRB1*04:05 positivity and PFS remained consistent with that in the main analysis, although statistical significance was not reached. These findings suggest that the observed association with PFS is unlikely to be solely explained by tumor type.

Although the difference in median PFS was modest, the consistent direction of the hazard ratios supports a potential association that warrants further investigation. Even a modest difference of approximately 1 month may be clinically meaningful in the context of advanced cancer, although this should be interpreted with caution.

This study focused on three pre-specified HLA-DRB1 alleles based on prior evidence of type 1 diabetes susceptibility and resistance; however, the relatively small sample size and lack of external validation necessitate cautious interpretation. Therefore, these findings should be considered exploratory. The association between DRB1*04:05 positivity and shorter PFS was consistent across analyses and should be interpreted in the context of effect size and confidence intervals rather than statistical significance alone.

Patients with HLA susceptibility to type 1 diabetes may be at increased risk of ICI-induced diabetes (25). We previously reported that β-cell function, assessed by HOMA-β, is associated with survival outcomes (4). ICIs can induce systemic immune activation that affects pancreatic β cells (26). Individuals with HLA alleles that enhance self-reactive CD4+ T-cell responses may have reduced tolerance to this immune stress (27). These mechanisms may link HLA genotype, β-cell vulnerability, and treatment outcomes.

In the present study, HbA1c levels were modestly elevated after ICI administration in the DRB1*09:01-positive group. Although ICI therapy can elicit autoimmune responses against pancreatic islets, no significant changes were observed in indices of insulin secretion or insulin resistance, including the C-peptide index, HOMA-β, and HOMA-IR. Therefore, these findings should be interpreted with caution, and the potential influence of HLA class II alleles on pancreatic β-cell function during immune stress remains uncertain.

This study had certain limitations. First, the sample size was relatively small and derived from a single-center cohort, which may have limited the statistical power and generalizability of the results. Second, all participants were Japanese, and the distribution of HLA alleles exhibited considerable variations among different ethnic groups. Therefore, the identified association between DRB1*04:05 and shorter PFS may not be directly applicable to populations with different genetic backgrounds. Third, the study included patients with various cancer types and treatment protocols. Although we adjusted for key clinical variables such as age, sex, BMI, ECOG performance status, cancer type, and treatment regimen, and performed sensitivity analyses in more homogeneous subsets, residual confounding attributable to tumor biology or treatment-related factors cannot be excluded. Another important limitation is that established biomarkers of ICI efficacy, including PD-L1 expression, tumor mutational burden (TMB), and microsatellite instability (MSI), were not available in this cohort. These factors are known to influence treatment response and survival outcomes and may have acted as unmeasured confounders. Therefore, the present findings should not be interpreted as demonstrating an independent causal relationship between DRB1*04:05 and ICI efficacy, but rather as an observational association. In addition, the proportional hazards assumption was not fully satisfied for some covariates (age and BMI), suggesting potential time-varying effects, which may have influenced the estimated hazard ratios; therefore, the results should be interpreted with caution. Finally, although the biological plausibility of HLA class II involvement in anti-tumor immunity is supported by previous studies, our analysis was observational and could not determine causality. Further studies with larger multiethnic cohorts that include mechanistic investigations are required to validate and expand these findings.

In summary, an association between HLA class II alleles and shorter PFS, and to a lesser extent OS, was observed in the context of ICI therapy. Furthermore, DRB1*04:05 may represent an immunogenetic marker associated with treatment outcomes in patients with advanced cancer, although validation in larger, independent cohorts along with further investigation into the underlying biological mechanisms is needed.

## Data Availability

The original contributions presented in the study are included in the article/**Supplementary Material**. Further inquiries can be directed to the corresponding author.
