# Supplementary material for: Association between HLA-DRB1*04:05 and the efficacy of immune checkpoint inhibitors for patients with advanced cancer
Source: Front Endocrinol (Lausanne). 2026 Jun 10;17:1789039. doi: 10.3389/fendo.2026.1789039 (PMC13290528; doi:10.3389/fendo.2026.1789039)
Supplement: Supplementary file 1 [file Table1.docx]

Supplemental Table 1. Changes in glucose tolerance before and after immune checkpoint inhibitor therapy according to the presence or absence of DRB1*09:01

|  | DRB1*09:01  negative (n=41) | DRB1*09:01  positive (n=11) | P-value |
| --- | --- | --- | --- |
| ΔHbA1c (%) | -0.10 (-0.30–0.19)  (n=58) | 0.15 (-0.20–0.30)  (n=14) | 0.035 |
| ΔCPI | 0.102 (-0.229–0.360) | -0.064 (-0.466–0.265) | 0.494 |
| ΔHOMA-β | -4.18 (-17.27–15.02) | 0.01 (-33.34–13.74) | 0.981 |
| ΔHOMA-IR | 0.120 (-0.416–0.480) | 0.178 (-0.682–0.884) | 0.906 |

Data are shown as median (25–75th percentile) for continuous variables.

The Mann–Whitney U test was applied for continuous variables

Δ: Difference between baseline and 1-month after ICI administration; HbA1c, glycated hemoglobin; CPI, C-peptide index; HOMA-β, homeostasis model assessment of beta cells; HOMA-IR, homeostasis model assessment of insulin resistance.

Supplemental Table 2. Changes in glucose tolerance before and after immune checkpoint inhibitor therapy according to the presence or absence of DRB1*04:05

|  | DRB1*04:05  negative (n=34) | DRB1*04:05  positive (n=18) | P-value |
| --- | --- | --- | --- |
| ΔHbA1c (%) | -0.05 (-0.75–0.20)  (n= 48) | -0.1 (-0.3–0.0)  (n= 24) | 0.269 |
| ΔCPI | 0.095 (-0.155–0.403) | 0.020 (-0.576–0.202) | 0.211 |
| ΔHOMA-β | -0.36 (-15.63–14.96) | -4.74 (-58.50–15.28) | 0.379 |
| ΔHOMA-IR | 0.14 (-0.30–0.49) | -0.19 (-0.55–0.57) | 0.379 |

Data are shown as median (25–75th percentile) for continuous variables.

The Mann–Whitney U test was applied for continuous variables

Δ: Difference between baseline and 1-month after ICI administration; HbA1c, glycated hemoglobin; CPI, C-peptide index; HOMA-β, homeostasis model assessment of beta cells; HOMA-IR, homeostasis model assessment of insulin resistance.

Supplemental Table 3. Changes in glucose tolerance before and after immune checkpoint inhibitor therapy according to the presence or absence of DRB1*15:01

|  | DRB1*15:01  negative (n=49) | DRB1*15:01  positive (n=3) | P-value |
| --- | --- | --- | --- |
| ΔHbA1c (%) | -1.0 (-0.30–0.10)  (n=67) | -0.10 (-0.30–0.15)  (n=5) | 0.932 |
| ΔCPI | 0.102 (-0.277–0.360) | 0.056 (-0.30–0.16) | 0.711 |
| ΔHOMA-β | -3.20 (-17.76–14.87) | -0.35 (-78.3–36.4) | 0.895 |
| ΔHOMA-IR | 0.123 (-0.435–0.512) | -0.026 (-0.44–0.15) | 0.581 |

Data are shown as median (25–75th percentile) for continuous variables.

For the DRB1*15:01-positive group (n=3), values are presented as median (range) due to the small sample size.

The Mann–Whitney U test was applied for continuous variables

Δ: Difference between baseline and 1-month after ICI administration; HbA1c, glycated hemoglobin; CPI, C-peptide index; HOMA-β, homeostasis model assessment of beta cells; HOMA-IR, homeostasis model assessment of insulin resistance.
